# What is the extent of reliability and validity evidence for screening tools for cognitive and behavioral change in people with ALS? A systematic review

**DOI:** 10.1080/21678421.2024.2314063

**Published:** 2024-02-28

**Authors:** Lyndsay Didcote, Silia Vitoratou, Ammar Al-Chalabi, Laura H. Goldstein

**Affiliations:** 1 Department of Psychology, Institute of Psychiatry, Psychology and Neuroscience, King’s College London, London, UK; 2 Department of Biostatistics and Health Informatics, Institute of Psychiatry, Psychology and Neuroscience, King’s College London, London, UK; 3 Department of Basic and Clinical Neuroscience, Maurice Wohl Clinical Neuroscience Institute, King’s College London, London, UK, and; 4 Department of Neurology, King’s College Hospital NHS Foundation Trust, London, UK

**Keywords:** Amyotrophic lateral sclerosis, cognition, behavior, screening tools, psychometrics

## Abstract

*Objective:* This systematic review provides an updated summary of the existing literature on the validity of screening tools for cognitive and behavioral impairment in people with Amyotrophic Lateral Sclerosis (pwALS), and also focuses on their reliability. *Method:* The following cognitive and behavioral screening tools were assessed in this review: the Edinburgh Cognitive and Behavioral ALS Screen (ECAS); the ALS Cognitive Behavioral Screen (ALS-CBS), the Mini Addenbrooke’s Cognitive Examination (Mini-ACE), the Beaumont Behavioral Interview (BBI); the MND Behavior Scale (MiND-B); and the ALS-FTD Questionnaire (ALS-FTD-Q). A search, using Medline, PsychINFO and Embase (21/09/2023), generated 37 results after exclusion criteria were applied. Evidence of internal consistency, item-total correlations, inter-rater reliability, clinical validity, convergent validity, and structural validity were extracted and assessed and risk of bias was evaluated. *Results:* The cognitive component of the ECAS was the tool with most evidence of reliability and validity for the assessment of cognitive impairment in ALS. It is well-suited to accommodate physical symptoms of ALS. For behavioral assessment, the BBI or ALS-FTD-Q had the most evidence of reliability and validity. The BBI is more thorough, but the ALS-FTD-Q is briefer. *Conclusions:* There is good but limited evidence for the reliability and validity of cognitive and behavioral screens. Further evidence of clinical and convergent validity would increase confidence in their clinical and research use.

## 1. Introduction

Around 50% of people with ALS (pwALS) show signs of cognitive or behavioral change, with 15% having ALS-frontotemporal dementia (ALS-FTD) (1, 2). Diagnostic criteria for cognitive impairment (ALSci), behavioral impairment (ALSbi), simultaneous cognitive and behavioral impairment (ALScbi), and ALS-FTD are outlined by the ALS-frontotemporal spectrum disorder (ALS-FTSD) criteria (3) and the consensus criteria for the diagnosis of behavioral variant FTD (bvFTD) (4). Several screening tools have been developed to detect changes to cognition and behavior in pwALS.

Previous systematic reviews of the reliability and validity of cognitive and behavioral screening tools for ALS have been conducted (5–7). A review of cognitive and behavioral screening tools (5) concluded that the cognitive component of the Edinburgh Cognitive and Behavioral ALS Screen (ECASc) (8) had strong clinical validity, though validity evidence of the behavioral component of the ECAS (ECASb) was limited. It was also concluded that the Beaumont Behavioral Interview (BBI) (9) assessed the widest range of behavioral impairment so was most promising but validation evidence was limited.

Another systematic review (7) concluded that the ECASc and cognitive component of the ALS Cognitive Behavior Scale (ALS-CBSc) (10) had good clinical validity. Studies in this review (7) demonstrated satisfactory clinical validity for the behavioral component of the ALS-CBS (ALS-CBSb), BBI, and MND Behavior Scale (MiND-B) (11) but highlighted that the MiND-B assesses a smaller number of behavioral domains than the other screens and may not fully assess the Rascovsky et al. (4) criteria for bvFTD.

Taule et al. (6) conducted a systematic review of reliability and validity evidence for the ECASc, ALS-CBSc and other non-ALS-specific measures (12–15). They reported that the ECAS and versions of the Addenbrooke’s Cognitive Examination (12, 13) were evaluated most for the greatest number of psychometric properties.

Gosselt et al. (7) and Simon & Goldstein (5) did not review evidence for the reliability of the cognitive screens, and Taule et al. (6) only evaluated reliability and validity for the ECAS and ALS-CBS. Taule et al. (6) made no assessment of behavioral screens other than the ALS-CBS. None of these three reviews assessed the reliability or validity of the Mini Addenbrooke’s Cognitive Examination (Mini-ACE) (16). While the Mini-ACE (16) is not an ALS-specific measure, it has been suggested as a suitable method of screening for cognitive change in ALS (17). Since Gosselt et al. (7), Simon & Goldstein (5), and Taule et al. (6) published their systematic reviews, further studies of the reliability and validity of the screening measures have emerged.

The aim of this systematic review was, therefore, to assess the extent of both current reliability and validity evidence for different screening tools for cognitive and behavioral change in pwALS including the Mini-ACE. The ALS-specific cognitive screening tools (i.e. screening tools designed specifically to detect cognitive changes likely to be found in pwALS) assessed in this systematic review were the ECASc and the ALS-CBSc; of note, however, the ECASc also assesses cognitive functions that are less likely to be observed in pwALS, referred to as ALS-non-specific scores, although only total ECASc scores were considered here. The ALS-specific behavioral screening tools that were assessed in this systematic review were the ECASb, the ALS-CBSb, the BBI, the MiND-B, and the ALS-FTD Questionnaire (ALS-FTD-Q) (18).

## 2. Methods

This systematic review was conducted according to Preferred Reporting Items for Systematic Reviews and Meta-Analyses (PRISMA) guidelines (19).

### 2.1. Eligibility

Articles in press, conference proceedings, abstracts, non-observational studies, papers not published in English, and other systematic reviews were excluded. Studies were excluded if they did not report reliability or validity statistics.

Participant samples could include pwALS or healthy controls. Reported statistics must have been based on screening tool total scores and not sub-section scores. Studies needed to have determined cutoff scores, evaluated cutoff scores based on their sensitivity and specificity, or evaluated a screen’s ability to differentiate between people with ALS and people with ALS with ALSci or ALSbi (e.g. using ROC curve analysis) to provide evidence of clinical validity.

Test-retest studies where test sessions were conducted more than two weeks apart or studies that did not report the test-retest interval were excluded (20). Test-retest studies that were conducted over a longer period may be influenced by declining cognition and behavior.

Demonstrating a significant difference in scores between ALSci or ALSbi and ALS with no impairment was deemed insufficient evidence of clinical validity (21, 22).

### 2.2. Search strategy

**Figure 1.**
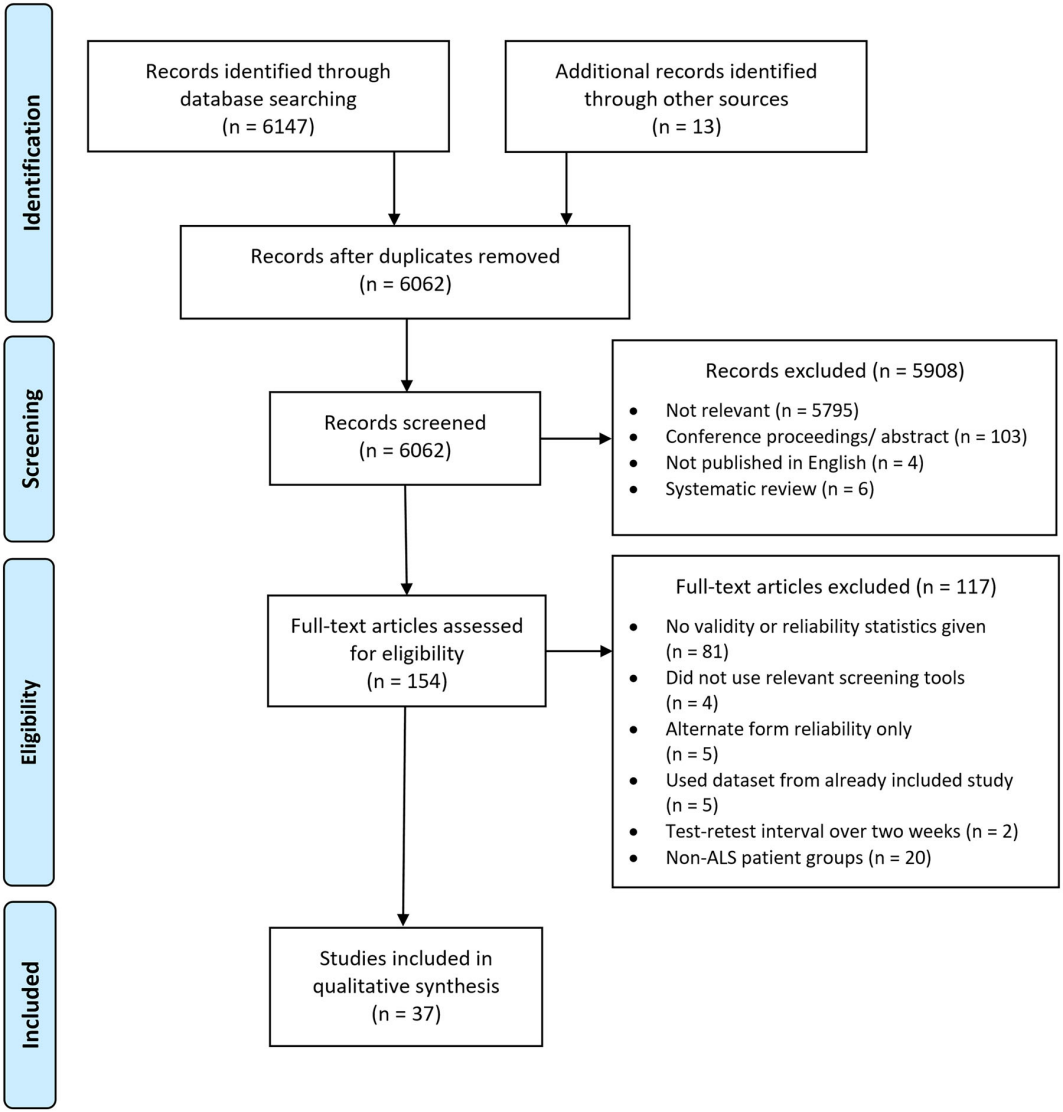
Prisma flow diagram.

The Embase, PsychINFO, and Medline databases were searched on 04/05/2022; searches were updated on 21/09/2023. The search terms are listed in Table 1. The process of article selection is detailed in Figure 1.

**Table 1.** Search terms for systematic review evaluating the reliability and validity of cognitive and behavioral screening tools.

Category	Search terms
Screening tools for cognitive/behavioral change	ECAS OR Edinburgh Cognitive and Behavio*ral ALS Screen OR Edinburgh Cognitive Behavio*ral ALS Screen OR Edinburgh Cognitive and Behavio*ral Amyotrophic Lateral Sclerosis Screen OR Edinburgh Cognitive Behavio*ral Amyotrophic Lateral Sclerosis Screen OR ALS-CBS OR ALS CBS OR Amyotrophic Lateral Sclerosis CBS OR ALS Cognitive Behavio*ral Screen OR Amyotrophic Lateral Sclerosis Cognitive Behavio*ral Screen OR Amyotrophic Lateral Sclerosis-CBS OR ALS-Cognitive Behavio*ral Screen OR Amyotrophic Lateral Sclerosis-Cognitive Behavio*ral Screen OR Mini-ACE OR Mini ACE OR Mini Addenbrooke’s Cognitive examination OR Mini-Addenbrooke’s Cognitive examination OR M-ACE OR MACE OR ALS-FTD-Q OR Amyotrophic Lateral Sclerosis-FTD-Q OR Amyotrophic Lateral Sclerosis FTD-Q OR Amyotrophic Lateral Sclerosis Frontotemporal Dementia Questionnaire OR Amyotrophic Lateral Sclerosis-Frontotemporal Dementia Questionnaire OR ALS Frontotemporal Dementia questionnaire OR ALS-Frontotemporal Dementia Questionnaire OR BBI OR Beaumont Behavio* Inventory, OR MiND-B OR Motor Neuron* Disease Behavio*r scale OR Motor Neuron* Disease-Behavio*r scale

### 2.3. Process of study selection

Titles and abstracts of records identified through electronic database searching were screened and duplicates were removed. Reference lists of included studies were hand searched for articles not found in the electronic literature search and 13 were identified. After exclusion criteria were applied, 37 articles remained (Figure 1). Where it was not clear whether a paper met exclusion criteria, three researchers (LD, SV, LHG) decided whether papers should be included in the review.

### 2.4. Data synthesis and extraction

No changes were made to the data extraction form following a pilot of search terms, exclusion criteria and data extraction. Information was extracted from articles by one researcher (LD). One study author was contacted for statistical clarification. Studies were grouped by screening tool. See Supplementary Material for further details on data synthesis and extraction.

### 2.5. Risk of bias and study quality

No established quality evaluation tool was used in this systematic review; instead, characteristics that were important to the types of study assessed in this review were evaluated. This permitted the tailoring of the assessment of risk of bias and study quality to the currently evaluated studies. One researcher (LD) made risk of bias and study quality assessments with input from two other researchers (SV and LHG).

A risk of bias and study quality score was assigned to studies, with high scores indicating a low risk of bias (good quality) and low scores indicating a high risk of bias (poor quality). Bias and study quality scores were calculated as total points awarded for each bias assessment criterion over total points available (not all bias assessment criteria were applicable to each study) and are given as a percentage. See Supplementary Material and table legends for details.

## 3. Results

Demographic data extracted from the included studies is presented in Table 2 and ALS data is presented in Table 3. Excluded studies are listed in Supplementary Material. Risk of bias and study quality (Tables 4 and 5) varied across studies.

**Table 2.** Demographic characteristics.

Measure	Author	Sample size (n)	Age Mean (sd)	Female (%)	Years of education Mean (sd)	Language
ECASc	Abrahams et al., 2014	C:40; ALS:48	ALS:60.0 (11.4); C:59.2 (12.6)	C:55.0; ALS:31.3	ALS:11.5 (2.2); C:12.3 (2.5)	English
ECASc	Lulé et al., 2015	ALS:136; C:60; IRR-C:10	ALS:60.0 (10.1); C:60.0 (12.6)	ALS:33.1; C:50.0	ALS:13.7 (2.9); C:13.9 (3.1)	Swiss-German
ECASc	Niven et al., 2015	ALS:40; C:40	ALS:64.5 (10.2); C:62.7 (10.5)	C:35.0; ALS:35.0	ALS:11.2 (2.0); C:12.3 (3.4)	English
ECAS	Poletti et al., 2016	ALS:107; C:248; IRR-C:30	ALS:63.0 (12.5); C:57.8 (10.6)	ALS:34.6; C:58.1	ALS:10.8 (4.2); C:14.1 (4.6)	Italian
ECASc	Ye et al., 2016	ALS:84; C:84	ALS:55.1 (10.7); C:54.8 (11.0)	ALS:31.0; C:32.1	ALS:11.5 (3.4); C:11.5 (3.4)	Chinese
ECASc	de Icaza Valenzuela et al., 2018	C:57	56.0 (13.3)	40.4	NR	English
ECASc	Bakker et al., 2019	ALS:428; C:428	ALS:63.7 (10.8); C:63.7 (10.8)	ALS:38.3; C:38.3	NR	Dutch
ECASc	Díaz et al., 2019	ALS:18	64.5 (11.6)*	45.0*	NR	Spanish
ECASc	Saxon et al., 2020	bvFTD:23; ALS-FTD:20; C:30	ALS-FTD:65.0 (8.0); bvFTD:60.0 (7.0); C:59.0 (8.0)	ALS-FTD:40.0; bvFTD:39.1; C:30.0	ALS-FTD:13.0 (3.0); bvFTD:12.0 (3.0); C:14.0 (3.0)	English
ECASc	Mojtabavi et al., 2021	ALS:30; C:31	ALS:53.9 (13.7); C:50.2 (11.5)	ALS:46.7; C:74.9	ALS:11.7 (3.6); C:14.0 (2.7)	Persian
ECASc	Fazio et al., 2021	C:102	54.9 (14.6)	52.9	14.5 (2.4)	Czech
ECASc	Beeldman et al., 2021	ALS:29	62.0 (8.7)	31.0	14.2 (2.6)	Dutch
ECASc	Watanabe et al., 2021	ALS:35; C:28	ALS:68.2 (7.4); C:63.8 (12.7)	ALS:42.9; C:42.9	ALS:11.9 (2.7); C:13.0 (2.4)	Japanese
ECASc	Aiello et al., 2022	ALS:89	63.1 (10.4)	43.8	11.7 (4.2)	Italian
ECASc	Albertyn et al., 2022	ALS:21; C:21; IRR-C:45	ALS:53.2 (13.4); C:53.9 (12.5)	ALS: 61.9; C:57.1	ALS:9.7 (2.8); C:10.2 (2.9)	Afrikaans; English
ECASc	Kacem et al., 2022	C:97; ALS:85	C:59.5 (11.7); ALS:59.5 (11.7)	C:40.2; ALS:31.4	NR	Tunisian Arabic
ECASc	Lee et al., 2023	ALS:38	61.3 (9.2)	50.0	12 (9-16)**	Korean
ECAS ALS-CBS	Mora et al., 2018	ALS:102; C:40; ALS-FTD:8	ALS:56.4 (NR); C:55.4 (NR); ALS-FTD:61.5 (NR)	ALS:36.3; C: 60.0; ALS-FTD:37.5	C:19.1 (NR); ALS:18.5 (NR); ALS-FTD:17.0 (NR)	Spanish
ECASc ALS-CBSc	Greco et al., 2021	ALS:154	66.3 (10.2)	46.1	NR	Italian
ECASc ALS-CBSc	Aiello, Solca, Greco, Torre, et al., 2023	ALS:293	64.6 (11.1)	48.1	11.6 (4.4)	Italian
ECAS ALS-CBS Mini-ACE	Kourtesis et al., 2019	C:52; ALS 28; AD:26	ALS:68.5 (7.1); AD:67.2 (4.1); C:NR	ALS:41.7; AD:46.2	ALS:11.1 (3.2); AD:11.1 (3.2)	Greek
ALS-CBS	Woolley et al., 2010	ALS:31; C:15	ALS:56.0 (NR); C:54.0 (NR)	ALS:45.0; C:67.0	ALS:14.5 (NR); C:14.0 (NR)	English
ALS-CBS	Turon-Sans et al., 2016	ALS:50	62.3 (NR)	36.0	NR	Spanish
ALS-CBS	Murphy et al., 2016	ALS:274	60.5 (10.1)	41.6	NR	English
ALS-CBSc	Branco et al., 2017	ALS:49; C:10	ALSci:60.3 (12.7); ALSni:55.1 (8.2); C:56.5 (7.7)	ALSci:40.0; ALSni:41.0; C:20.0	ALSci:7.5 (3.4); ALSni:9.9 (5.2); C:11.8 (3.8)	Portuguese
ALS-CBSc	Tremolizzo et al., 2020	C:458; ALS:100	C:56.4 (16.8); ALS:64.3 (10.2)	ALS:33.0	C:11.4 (4.7); ALS:10.1 (4.2)	Italian
ALS-CBSb ECASb	Aiello, Solca, Greco, La Tona, et al., 2023	ALS:265	65.0 (10.9)	47.9	11.4 (4.2)	Italian
ALS-CBSb	Murphy et al., 2015	ALS:24	59.6 (1.8)	38.0	14.6 (1.7)	English
ALS-FTD-Q	Raaphorst et al., 2012	ALS:103; ALS-bvFTD:10; bvFTD:25; MC:39; C:31	ALS:61.4 (11.9); ALS-bvFTD:60.2 (10.4); bvFTD:63.4 (6.2); MC:58.7 (13.3); C:58.3 (11.7)	ALS:29.1; ALS-bvFTD:30.0; bvFTD:32.0; MC:43.6; C:51.6	NR	Dutch
ALS-FTD-Q	Watanabe et al., 2016	ALS:92; ALS-bvFTD:6; bvFTD:16; C:32	ALS:67.7 (10.2); ALS-bvFTD:67.2 (6.9); bvFTD:67.1 (8.4); C:62.1 (8.6)	ALS:40.2; ALS-bvFTD: 50.0; bvFTD:37.5; C:56.3	NR	Japanese
ALS-FTD-Q BBI	Pinto-Grau et al., 2017	ALS:60	65.4 (9.7)	30.0	13.2 (3.4)	English
BBI	Elamin et al., 2017	ALS:85; C:78	ALS:63.1 (12.3); C:63.2 (9.6)	ALS:32.9; C:47.4	ALS:13.3 (3.4); C:14.1 (3.9)	English
BBI	Burke et al., 2017	ALS:317; C:66	ALS:63.0 (11.1); C:61.4 (13.7)	ALS:49.0; C:55.0	ALS:12.9 (3.8); C:13.5 (3.8)	English
BBI	Iazzolino et al., 2022	ALS:90; C:100	ALS:63.3 (10.6); C:62.3 (11.7)	ALS:56.7; C:51.0	ALS:11.7 (4.2); C:12.2 (3.9)	Italian
MiND-B	Mioshi et al., 2014	ALS:79	limb onset: 60.4 (11.6); bulbar onset: 58.7 (10.4)	limb onset:40.0; bulbar onset:54.0	limb onset:13.7 (3.2); bulbar onset:14.1 (2.9)	English
MiND-B ECASc	Rahimzadeh Goradel et al., 2023	ALS:46	54.9 (10.3)	32.6	NR	Persian
MiND-B Mini-ACE	Hsieh, Caga, et al., 2016	ALS:70; ALSPlus:19; ALS-FTD:24; C:45	ALS:56.3 (11.8); ALSPlus:57.6 (10.3); ALS-FTD:63.1 (8.9); C:67.6 (5.9)	ALS:40.7; ALSPlus:36.8; ALS-FTD:33.3; C:48.9	ALS:13.5 (3.1); ALSPlus:12.5 (2.9); ALS-FTD:12.3 (3.1); C:13.1 (2.9)	English

Note: C = control. MC = muscle disease control. ALSci = ALS with cognitive impairment. ALSni = ALS with no impairment. bvFTD = behavioral variant frontotemporal dementia. ECASc = ECAS cognitive section. ALS-CBSc = ALS-CBS cognitive section. NR = Not reported. * = these statistics are representative of the wider sample of 40 ALS participants. IRR-C = healthy controls studied to determine inter-rater reliability. * Language refers to the language in which the screening tool was delivered. ** = Median (IQR) is reported in place of mean (sd).

**Table 3.** ALS sample characteristics.

Measure	Author	Disease duration (months) Mean (sd)	ALSFRS-R Mean (sd)	Onset site (%)
ECASc	Abrahams et al., 2014	25.0 (NR)**	25.2 (11.4)	Bulbar: 29.2
ECASc	Lulé et al., 2015	39.0 (41.4)	34.9 (7.8)	Spinal: 80.2; Bulbar: 19.9
ECASc	Niven et al., 2015	25.5 (NR)**	35.7 (NR)	NR
ECAS	Poletti et al., 2016	21.1 (19.4)	37.2 (6.8)	Bulbar: 21.5; Limb: 76.6
ECASc	Ye et al., 2016	15.8 (14.5)	41.3 (4.6)	Bulbar: 19.1; Thoracic: 3.6; Cervical: 58.3; Lumbar: 19.1
ECASc	Bakker et al., 2019	NR	37.6 (7.1)	Spinal: 72.2; Bulbar: 26.6
ECASc	Díaz et al., 2019	65.6 (54.8)*	25.2 (11.4)*	Spinal: 67.5; Bulbar: 32.5*
ECASc	Saxon et al., 2020	36.0 (3.0)	NR	NR
ECASc	Mojtabavi et al., 2021	NR	35.9 (7.2)	NR
ECASc	Beeldman et al., 2021	9.0 (NR)**	40.0 (NR)**	Limb: 72.4; Bulbar: 20.7; Mixed: 7.0
ECASc	Watanabe et al., 2021	55.3 (NR)**	33.3 (10.1)	Bulbar: 25.7; Cervical: 37.1; Lumbar: 37.1
ECASc	Aiello et al., 2022	NR	39.9 (4.7)	NR
ECASc	Albertyn et al., 2022	NR	35.8 (7.4)	Spinal: 76.2; Bulbar: 23.8
ECASc	Kacem et al., 2022	NR	38.3 (8.7)	Spinal: 72.1; Bulbar 19.8
ECASc	Lee et al., 2023	17.5 (10-31)**	37.5 (5.9)	Limb:71.1; Bulbar:28.9
ECAS ALS-CBS	Mora et al., 2018	ALS:40.5 (NR)**; ALS-FTD: 20.7 (NR)**	NR	ALS – Limb: 81.9; Bulbar: 13.8; Respiratory and other: 1.1; Unknown: 3.2 ALS-FTD – Limb: 75.0; Bulbar: 25.0
ECASc ALS-CBSc	Greco et al., 2021	56.1 (75.7)	28.0 (9.6)	Bulbar: 26.6; Spinal: 66.2; Generalized: 4.1
ALS-CBSc ECASc	Aiello, Solca, Greco, Torre, et al., 2023	21.9 (36.6)	33.1 (10.1)	NR
ECAS ALS-CBS Mini-ACE	Kourtesis et al., 2019	NR	NR	NR
ALS-CBS	Woolley et al., 2010	22.0 (NR)	34.0 (NR)	Limb: 66.0; Bulbar: 34.0
ALS-CBS	Turon-Sans et al., 2016	18.0 (NR)	29.2 (NR)	Spinal: 76.0; Bulbar: 22.0
ALS-CBS	Murphy et al., 2016	11.8 (4.5)	36.1 (6.6)	Bulbar: 32.1; Cervical: 32.5; Thoracic: 1.1; Lumbosacral: 32.1; Respiratory: 1.8; Other: 0.4
ALS-CBSc	Branco et al., 2017	ALSci:25.8 (29.7); ALSni:39.8 (52.7)	ALSci:35.3 (5.3); ALSni:35.0 (7.5)	NR
ALS-CBSc	Tremolizzo et al., 2020	42.0 (37.0)	31.2 (9.2)	Spinal: 81.0; Bulbar: 19.0
ALS-CBSb ECASb	Aiello, Solca, Greco, La Tona, et al., 2023	38.7 (52.7)	32.4 (10.1)	NR
ALS-CBSb	Murphy et al., 2015	56.0 (49.0)	28.0 (1.9)	NR
ALS-FTD-Q	Raaphorst et al., 2012	ALS:33.5 (NR)**; ALS-bvFTD:35.5 (NR)**	ALS:31.5 (9.2); ALS-bvFTD:32.9 (6.1)	ALS & ALS-bvFTD – Limb: 82.3
ALS-FTD-Q	Watanabe et al., 2016	ALS:21.0 (NR)**; ALS-bvFTD:30.0 (NR)**	ALS:33.7 (9.7); ALS-bvFTD:26.8 (8.2)	ALS – Limb: 75.0; Bulbar: 25.0 ALS-bvFTD - Limb: 50.0; Bulbar: 50.0
ALS-FTD-Q	Pinto-Grau et al., 2017	NR	33.4 (6.2)	Spinal: 64.0; Bulbar: 28.0; Respiratory: 8.0
BBI	Elamin et al., 2017	NR	37.7 (6.4)	NR
BBI	Burke et al., 2017	22.1 (16.1)	35.2 (7.9)	Spinal: 56.7; Bulbar: 23.2; Respiratory: 2.9
BBI	Iazzolino et al., 2022	NR	39.9 (4.7)	NR
MiND-B	Mioshi et al., 2014	limb onset: 27.6 (28.0); bulbar onset: 15.2 (18.0)	limb onset: 30.5 (13.8); bulbar onset: 31.7 (13.5)	Limb: 69.6; Bulbar: 30.4
MiND-B ECASc	Rahimzadeh Goradel et al., 2023	NR	32.0 (10.4)	NR
MiND-B Mini-ACE	Hsieh, Caga, et al., 2016	ALS:18.0 (14.2); ALSPlus:28.9 (21.2); ALS-FTD:40.1 (26.3)	ALS:40.3 (6.9); ALSPlus:38.4 (6.4); ALS-FTD:37.9 (5.7)	ALS - Bulbar: 88.9; Limb: 11.1 ALSPlus – Bulbar: 36.8; Limb: 63.2 ALS-FTD – Bulbar: 66.7; Limb: 33.3

Note: ALSci = ALS with cognitive impairment. ALSni = ALS with no impairment. FTD = frontotemporal dementia. bvFTD = behavioral variant FTD. NR = not reported. ALSPlus = ALS with cognitive and/or behavioral impairment. ECASc = ECAS cognitive section. ALS-CBSc = ALS-CBS cognitive section. NA = not applicable. * = these statistics are representative of the wider sample of 40 ALS participants, rather than the sub-set of 18 participants for which validation statistics were generated. ** = Median (IQR) is reported in place of mean (sd).

**Table 4.** Risk of bias and study quality assessment.

Measure	Author	N ≥ 30 (0–1)*	ALS cohort (0-1)**	Validated against gold standard (0–2)***	Bias/quality score (% of available total)
ECASc	Abrahams et al., 2014	1	1	0	50
ECASc	Lulé et al., 2015	1	1	NA	100
ECASc	Niven et al., 2015	1	1	2	100
ECAS	Poletti et al., 2016	1	1	NA	100
ECASc	Ye et al., 2016	1	1	0	50
ECASc	de Icaza Valenzuela et al., 2018	1	0	NA	50
ECASc	Bakker et al., 2019	1	1	NA	100
ECASc	Díaz et al., 2019	0	1	NA	50
ECASc	Saxon et al., 2020	1	1	NA	100
ECASc	Mojtabavi et al., 2021	1	1	1	75
ECASc	Fazio et al., 2021	1	0	0	25
ECASc	Beeldman et al., 2021	0	1	2	75
ECASc	Watanabe et al., 2021	1	1	0	50
ECASc	Aiello et al., 2022	1	1	2	100
ECASc	Albertyn et al., 2022	0	1	NA	50
ECASc	Kacem et al., 2022	1	1	NA	100
ECASc	Lee et al., 2023	1	1	NA	100
ECAS ALS-CBS	Mora et al., 2018	1	1	0	50
ECASc ALS-CBSc	Greco et al., 2021	1	1	NA	100
ALS-CBSc ECASc	Aiello, Solca, Greco, Torre, et al., 2023	1	1	NA	100
ECAS ALS-CBS Mini-ACE	Kourtesis et al., 2019	1	1	2	100
ALS-CBS	Woolley et al., 2010	1	1	2	100
ALS-CBS	Turon-Sans et al., 2016	1	1	2	100
ALS-CBS	Murphy et al., 2016	1	1	NA	100
ALS-CBSc	Branco et al., 2017	1	1	2	100
ALS-CBSc	Tremolizzo et al., 2020	1	1	NA	100
ALS-CBSb ECASb	Aiello et al., 2023	1	1	NA	100
ALS-CBSb	Murphy et al., 2015	0	1	NA	50
ALS-FTD-Q	Raaphorst et al., 2012	1	1	NA	100
ALS-FTD-Q	Watanabe et al., 2016	1	1	NA	100
ALS-FTD-Q BBI	Pinto-Grau et al., 2017	1	1	NA	100
BBI	Elamin et al., 2017	1	1	1	75
BBI	Burke et al., 2017	1	1	NA	100
BBI	Iazzolino et al., 2022	1	1	2	100
MiND-B	Mioshi et al., 2014	1	1	2	100
MiND-B ECASc	Rahimzadeh Goradel et al., 2023	1	1	NA	100
MiND-B Mini-ACE	Hsieh, Caga, et al., 2016	1	1	2	100

Note: NA = not applicable as ROC curves were not generated and so gold standard classifications of impairment were not needed. ECASc = ECAS cognitive section. ALS-CBSc = ALS-CBS cognitive section. * *N* ≥ 30 was awarded one point. **Those using an ALS cohort were awarded one point. *** If validated against a gold standard of clinical diagnosis or outcome of a full neuropsychological test battery, two points were awarded; if validated against a widely accepted cognitive/behavioral measure, one point was awarded.

**Table 5.** Risk of bias and study quality assessment for inter-rater reliability assessments.

Measure	Author	N ≥ 30 (0–1)*	ALS cohort (0–1)**	Blinding (0–1)***	Randomly selected (0–1)****	Bias/quality score (% of available total)
ECASc	Lulé et al., 2015	0	0	1	0	25
ECASc	Poletti et al., 2016	1	0	1	1	75
ECASc	Albertyn et al., 2022	1	0	0	NAµ	33
ECASc	Kourtesis et al., 2019	1	1	1	NA	100

Note: µ = not relevant because whole sample was used or a separate additional sample was used. NA = not applicable. ECASc = ECAS cognitive section. *N ≥ 30 was awarded one point. **Those using an ALS cohort were awarded one point. ***If raters were blinded to scores given by other raters, studies were awarded one point. ****Where a sub-sample was selected from the larger main study sample, one point was awarded if the sub-sample was randomly selected.

### 3.1. Reliability

Eighteen of the 37 studies assessed internal consistency (see Table 6).

**Table 6.** Internal consistency.

Measure	Author	Cronbach’s alpha
ECASc	Abrahams et al., 2014	ALS & C: 0.75; ALS: 0.77
ECASc	Poletti et al., 2016	0.86
ECASc	Ye et al., 2016	0.74
ECAS	Mora et al., 2018	ECASc: 0.78; ECASb: 0.56
ECASc	Kourtesis et al., 2019	0.84
ECASc	Mojtabavi et al., 2021	ALS & C: 0.79; ALS: 0.78
ECASc	Fazio et al., 2021	0.69
ECASc	Watanabe et al., 2021	0.77
ECASc	Kacem et al., 2022	0.85/0.87**
ECASc	Lee et al., 2023	0.87
ALS-CBSb	Aiello, Solca, Greco, La Tona, et al., 2023	0.90*
ALS-FTD-Q	Raaphorst et al., 2012	0.92
ALS-FTD-Q	Watanabe et al., 2016	0.92
BBI	Elamin et al., 2017	0.89
BBI	Burke et al., 2017	0.91
BBI	Iazzolino et al., 2022	0.93
MiND-B	Mioshi et al., 2014	0.97
MiND-B	Rahimzadeh Goradel et al., 2023	0.70

Note: Papers omitted from this table did not report internal consistency of the relevant screening tools. ECASc = ECAS cognitive section. *Internal reliability based on McDonald’s ω, rather than Cronbach’s α. C = control group. **Both statistics were reported in different places in the paper but the corresponding author did not respond to a request for clarification.

There were nine studies showing acceptable internal consistency for the ECASc (8, 23–30), while one did not demonstrate acceptable internal consistency (31) (Table 6). This latter study (31) had a lower study bias/quality score (Table 4) than all other studies that reported acceptable internal consistency for the ECASc. The ECASc has been shown to have fair to good inter-rater reliability (26, 32, 33). Studies that reported better inter-rater reliability for the ECASc had higher bias/quality scores (see Table 5). There was no evidence of test-retest reliability for the ECASc within the parameters set in this systematic review; while Kacem et al. (29) reported test-retest statistics, their methodology and reported results did not meet inclusion criteria. Item-total correlations were unavailable for the ECASc.

Inter-rater reliability, test-retest reliability, internal consistency, and item-total correlations of the ALS-CBSc were not reported in any of the studies, within the parameters set in this systematic review. Similarly, inter-rater reliability, test-retest reliability, item-total correlation, and internal consistency for the Mini-ACE were not reported in the included studies.

The internal consistency of the ECASb was questionable as it was only assessed by one study (25) that found its internal consistency to be statistically unacceptable. While inter-rater reliability and test-retest reliability for the ALS-CBSb were not reported in any of the studies included here, one study suggested that the ALS-CBSb had acceptable internal consistency (34).

The ALS-FTD-Q had acceptable internal consistency and 80% of items had acceptable item-total correlations (18,28) (Table 6). Inter-rater reliability and test-retest reliability of the ALS-FTD-Q were not reported in any of the included studies.

While inter-rater reliability, test-retest reliability, and item-total correlations of the BBI were not reported, three studies reported that the internal consistency of the BBI was acceptable (9,35,36) (Table 6).

One study reported that 7/9 MiND-B items had acceptable item-total correlations (37) and two studies reported acceptable internal consistency (11, 37). The inter-rater reliability and test-retest reliability of the MiND-B were not reported in any of the studies.

### 3.2. Validity

Clinical and convergent validity of the different measures are summarized in Table 7. No validity data for the ALS-FTD-Q cutoff score has been reported.

**Table 7.** Test cutoff scores, sensitivity, specificity, area under the curve and convergent validity.

Measure	Author	Cutoff (sensitivity; specificity)	AUC	Convergent validity
ECASc	Abrahams et al., 2014	105		
ECASc	Lulé et al., 2015			ECASc/FAB: r = 0.53; ECASc/MoCA: r = 0.60
ECASc	Niven et al., 2015	^‡^105 (69; 89); 110 (92; 81)	0.91	
ECAS	Poletti et al., 2016			ECASc/MoCA: r = 0.70; ECASc/FAB: r = 0.63; ECASb/FBI: r = 0.63
ECASc	Ye et al., 2016	81.9		
ECASc	de Icaza Valenzuela et al., 2018			ECASc/ACE-III: r = 0.54
ECASc	Díaz et al., 2019			Memory component of neuropsychological battery / ECASc: Cohen’s kappa = 0.75
ECASc	Saxon et al., 2020			ECASc naming/ Graded Naming Test: r_s_ = 0.65; ECASc spelling/ PALPA spelling: r_s_ = 0.86; ECASc sentence completion/ Hayling test: r_s_ = 0.68; ECASc social cognition/ Judgment of Preference: r_s_ = 0.64
ECASc	Mojtabavi et al., 2021	88.6 (100; 50)	0.87	ECASc/MoCA: r_s_ = 0.74
ECASc	Fazio et al., 2021	86		
ECASc	Beeldman et al., 2021	105* (83; 83)	0.90	
ECASc	Watanabe et al., 2021	86.8		ECASc/MoCA: r_s_ = 0.53; ECASc/FAB: r_s_ = 0.59
ECASc	Aiello et al., 2022	** (74; 89)	** 0.87	
ECASc	Albertyn et al., 2022			ECASc/MoCA: r = 0.59
ECASc	Kacem et al., 2022			ECASc/FAB: r_s_ = 0.69; ECASc/MMSE: r_s_ = 0.72
ECASc	Lee et al., 2023			ECASc/MoCA: r = 0.69 ECASc/FAB: r = 0.55
ECAS ALS-CBS	Mora et al., 2018	ECASc: 73	ECASc: 0.75	ECASc/ALS-CBSc: r_s_ = 0.76; ECASb/ALS-CBSb: r_s_ = −0.58
ECASc ALS-CBSc	Greco et al., 2021			ECASc/ALS-CBSc: r_s_ = 0.64, Cohen’s kappa = 0.20
ECASc ALS-CBSc	Aiello, Solca, Greco, Torre, et al., 2023			ECASc/ALS-CBSc: R^2^: 0.71
ECAS ALS-CBS Mini-ACE	Kourtesis et al., 2019	ECASc AD from ALS: 93 (92; 55); Mini-ACE AD from ALS: 23 (97; 71)	ECASc AD from ALS: 0.85; Mini-ACE AD from ALS: 0.87	ALS-CBSc/ECASc: r = 0.82; ALS-CBSc/Mini-ACE: r = 0.73; ALS-CBSb/ECASb: r = −0.75
ALS-CBS	Woolley et al., 2010	ALS-CBSc ALSci: 17 (85; 71); ALS-FTD: 10 (100; 100); ALS-CBSb ALSbi: 36 (90; 86); ALS-FTD: 32 (88; 80)		
ALS-CBS	Turon-Sans et al., 2016	ALS-CBSc ALSci: 15 (86; 62); ALS-FTD: 8 (83; 75) ALS-CBSb ALSbi: 36 (93; 75); ALS-FTD: 35 (83; 69)	ALS-CBSc ALSci: 0.85; ALS-FTD: 0.87 ALS-CBSb ALSbi: 0.83; ALS-FTD: 0.81	
ALS-CBS	Murphy et al., 2016			ALS CBSb/FBI-ALSb: r_s_ = 0.72; ALS CBSc/FBI-ALSc: r_s_ = 0.49
ALS-CBSc	Branco et al., 2017	ALSci: 10 (90; 87)	0.91	
ALS-CBSc	Tremolizzo et al., 2020			ALS-CBSc/FAB: Cohen’s kappa = 0.55, r_s_ = 0.60; ALS-CBSc/WST: Cohen’s kappa= 0.60, r_s_ = 0.59
ALS-CBSb ECASb	Aiello, Solca, Greco, La Tona, et al., 2023			ALS-CBSb/FBI: r_s_ = −0.80; ALS-CBSb/ECASb: r_s_ = −0.59
ALS-CBSb	Murphy et al., 2015			ALS-CBSb/FBI-ALS negative subscale r = − 0.90; ALS-CBSb/FBI-ALS disinhibition subscale: r = − 0.82
ALS-FTD-Q	Raaphorst et al., 2012			ALS-FTD-Q/FrSBe: r_s_ = 0.80; ALS-FTD-Q/FBI: r_s_ = 0.79
ALS-FTD-Q	Watanabe et al., 2016			ALS-FTD-Q/FBI: r_s_ = 0.79
ALS-FTD-Q BBI	Pinto-Grau et al., 2017			BBI/ALS-FTD-Q: r_s_ = 0.81
BBI	Elamin et al., 2017	Mild: 7 (88; 79); Moderate: 22.5 (90; 96)	0.96	BBI/FrSBe: r = 0.76; BBI/FAB: r = 0.44
BBI	Iazzolino et al., 2022	11.5 (92; 78)	0.90	
MiND-B	Mioshi et al., 2014	35 (90; 50); 33 (81; 75)		
MiND-B ECASc	Rahimzadeh Goradel et al., 2023			MiND-B/ECASc: r = −.435^†^
MiND-B Mini-ACE	Hsieh, Caga, et al., 2016	MiND-B: 33* (90; 79) Mini-ACE: 25* (65; 90)		

Note: Papers omitted from this table did not report validity statistics of relevant screening tools. AUC = Area under the curve. * = used published cutoff score. ** = cutoff scores varied for age and education groups. ^†^ = Authors demonstrated divergent validity, that the MiND-B measures behavior, a concept that is separate to cognition (measured by the ECASc). ^‡^ = Niven et al. (2015) proposed an ECASc cognitive impairment cutoff score of between 105 and 110. r = Pearson’s correlation coefficient. r_s_ = Spearman’s rho. AD: Alzheimer’s disease. ALSci – ALS with cognitive impairment. ALSbi – ALS with behavioral impairment. ALS FTD – ALS with frontotemporal dementia. ECASc = ECAS cognitive section. ALS-CBSc = ALS-CBS cognitive section. ACE-III = Addenbrooke’s Cognitive Examination (58). FrSBe = Frontal Systems Behavior Scale (53). FBI = Frontal Behavioral Inventory (54). FBI-ALS = Frontal Behavioral Inventory ALS version (59). FBI-ALSc - cognitive component of FBI-ALS. FBI-ALSb = behavioral component of FBI-ALS. MoCA = Montreal Cognitive Assessment (15). FAB = Frontal Assessment Battery (14). WST = Weigl sorting test (60). PALPA = Psycholinguistic Assessments of Language Processing in Aphasia (61). Sensitivity and specificity scores of 1.0 are described as perfect; 0.90-0.99 are excellent; 0.80-0.89 are very good; 0.70-0.79 are good; 0.60-0.69 are fair; and 0.50-0.59 are medium; this descriptive system was generated for this systematic review.

#### 3.2.1. Clinical validity data for the ECASc

Generally, the sensitivity and specificity of ECASc cutoff scores varied from 0.5 to 1.0 (26, 27, 38–40); see Table 7. Based on ROC curve analyses, the ECASc was good (41) at discriminating between ALSci and cognitively normal individuals (27, 38, 40) and between ALSci and Alzheimer’s dementia (26).

Overall, there is substantial evidence that the ECASc has good clinical validity.

#### 3.2.2. Clinical validity data for the ALS-CBSc

For the ALS-CBSc, nine studies reported validity statistics (10,25,42–47) (Table 7). Three studies with good methodological quality examined the clinical validity of the ALS-CBSc (10,43,47) (Table 7). There were some differences in the cutoff scores identified by the different studies but the reported sensitivity and specificity of those cutoff scores were very good to perfect.

#### 3.2.3. Clinical validity data for the Mini-ACE

Validity data was given for the Mini-ACE by two studies (17,26) (Table 7). For differentiating Alzheimer’s dementia from pwALS, a cutoff slightly lower than the published cutoff score was identified, with excellent sensitivity and good specificity (26). For identifying ALS plus (having cognitive and/or behavioral symptoms) from ALS cognitively normal, the published cutoff had fair sensitivity and excellent specificity (17).

#### 3.2.4. Clinical validity data for the ALS-CBSb

Six papers reported ALS-CBSb validity data (10,26,34,45,47,48) (Table 7). Two studies supported the clinical validity of the ALS-CBSb (10, 47). They were in agreement over the cutoff score for determining ALS-FTD and reported similar cutoff scores for ALSbi, all with very good to excellent sensitivity and fair to very good specificity.

#### 3.2.5. Clinical validity data for the BBI

The two studies that reported clinical validity and were high in methodological quality identified different cutoff scores with very good to excellent sensitivity and good to excellent specificity (9, 35).

#### 3.2.6. Clinical validity data for the MiND-B

The published cutoff scores for the MiND-B had very good to excellent sensitivity and medium to good specificity, according to two studies with high methodological quality (11,17) (Table 7).

#### 3.2.7. Clinical validity data for the ALSFTD-Q

There were no available studies to support the clinical validity of the ALS-FTD-Q.

#### 3.2.8. Convergent validity (Table 7)

Convergent validity was demonstrated between the ECASc and the ALS-CBSc (25, 26, 42, 44), between the ECASc and Montreal Cognitive Assessment (MoCA) (15, 23, 30, 32, 33, 49) between the ECASc and the Frontal Assessment Battery (FAB) (14, 23, 29, 30, 33, 49), and between the ECASc and other cognitive tests (30, 50, 51). Evidence of the convergent validity of the ECASc was, therefore, very strong.

As well as evidence of convergent validity between the ECASc and ALS-CBSc (25, 26, 42, 44), there was also evidence of convergent validity between the ALS-CBSc and the Mini-ACE (26). In addition, there was evidence of convergent validity between the ALS-CBSc and other cognitive tests (45, 46).

There was evidence of convergent validity between the ECASb and ALS-CBSb (25,26,34) (Table 7) and between the ALS-CBSb and the other behavioral measures (34, 45, 48).

As well as the evidence of convergent validity between the ALS-FTD-Q and the BBI (52), convergent validity was demonstrated between the ALS-FTD-Q and the Frontal Systems Behavior Scale (FrSBe) (18, 53), and between the ALS-FTD-Q and the Frontal Behavioral Inventory (FBI) (18,49,54) (Table 7). There was evidence of convergent validity between the BBI and the FrSBe and between the BBI and the FAB but this evidence was not extensive (9) (Table 7). There was evidence of divergent validity between the MiND-B and the ECASc, suggesting that the MiND-B measures behavioral change as an independent construct from cognitive change (37).

#### 3.2.9. Structural validity

One study (34), using Principal Components Analysis (PCA), reported that the ALS-CBSb has a single component structure. For the BBI, another study (35) reported that five items loaded weakly on the component of behavioral impairment when using PCA but reported a clear single factor structure. When PCA was conducted for the MiND-B to confirm unidimensionality, only 44% of raw variance was explained (target: 50%) (11). Both studies were awarded high scores in the risk of bias and study quality assessment. The structural validity of the ECASc, ALS-CBSc, Mini-ACE, ECASb, and ALS-FTD-Q has not been assessed.

## 4. Discussion

The purpose of this systematic review was to assess the evidence of reliability and validity relating to screening tools for cognitive and behavioral change in pwALS. This systematic review assessed 37 studies. Most (20/37) studies focused on the ECASc.

### 4.1. Cognitive screens

For cognitive screening tools, the methodological quality of studies varied, with 17/26 studies achieving the maximum possible score (signifying low risk of bias and high study quality).

Twenty studies reported reliability and/or validity statistics for the ECASc (Tables 6 and 7).

Given the extensive evidence of the reliability (internal consistency and inter-rater reliability) and validity of the ECASc and given that it is ALS-specific and tailored to people with ALS who are unable to either speak or write a response, the ECASc appears to be a suitable assessment option for clinicians and researchers, based on this review.

Ten studies reported validity statistics for the ALS-CBSc (Table 7). Overall, there was good evidence for the validity of the ALS-CBSc, though evidence of its reliability was limited. The ALS-CBSc is an ALS-specific measure, but there are insufficient adjustment mechanisms in place for people with ALS who cannot write or cannot say their responses. Despite good evidence of validity, the ALS-CBSc is limited by its lack of accessibility for some pwALS.

Evidence of the validity of Mini-ACE, in terms of its use with pwALS, was limited and there was no data concerning the Mini-ACE’s reliability. The Mini-ACE is also not ALS-specific and makes no adjustments for writing/speech difficulties. Based on the evidence considered in this systematic review, the Mini-ACE was the weakest of the three cognitive screening tools for identifying cognitive impairment in pwALS.

### 4.2. Behavioral screens

The methodological quality of behavioral screen studies was generally high. Reliability and validity statistics were presented for the ECASb by only four studies (23,25,26,34) (Tables 6 and 7). The internal consistency of the ECASb was questionable as it was only assessed by one study (25) that found its internal consistency to be statistically unacceptable. However, this may not necessarily be a weakness of the ECASb since there is no published evidence for its unidimensionality and no indication that identified behaviors contribute equally to a classification of ALSbi. Evidence of validity and reliability for the ECASb was, therefore, limited, thereby reducing evidence that can be used to justify its choice for the behavioral assessment of people with ALS.

There was good evidence for the validity of the ALS-CBSb but this was not extensive and there was limited evidence of ALS-CBSb reliability.

While evidence of the validity and reliability of the ALS-FTD-Q comes from a small number of studies, the quality of evidence was good and is, therefore, a good option for clinicians and researchers.

For the BBI, validity and reliability statistics were reported by four studies (9,35,36,52) (Tables 6 and 7). The BBI is ALS-specific and very thorough, though lengthy. The evidence reviewed in this study suggests that the BBI is a good option for researchers and clinicians who are not under assessment time constraints.

Three studies evaluated the reliability and validity of the MiND-B (11,17,37) (Tables 6 and 7). Due to limited evidence of the reliability and validity of the MiND-B evaluated in this review, it is likely not the best option for clinicians and researchers, especially given that other measures are available.

Consistent with a previous review (5), the current review suggested that the ECASc was the most appropriate screening tool for cognitive impairment in ALS with the strongest validation evidence. Another review (7) also concluded that the ECASc, along with the ALS-CBSc, had evidence of good clinical validity. A third review (6), however, highlighted that assessments of the psychometric properties of the ALS-CBSc are limited. The findings of this systematic review do not contradict this statement, but this is true of all of the assessed cognitive and behavioral screening tools, with the exception of the ECASc. It was previously highlighted (5) that the evidence of behavioral screening tool validity was limited; this is still the case, although eight studies have since contributed to the evidence-base (25, 26, 29, 30, 34, 35, 37, 42). The results of the current review concur with previous conclusions (5) that the validity of the BBI and ALS-FTD-Q is similar but as the BBI is more comprehensive, it may be the better option. The advantage of the ALS-FTD-Q is that it is briefer and therefore quicker to complete than the BBI.

This systematic review assessed most ALS-specific screening tools for cognitive and behavioral impairment. This is the first systematic review of several ALS-specific cognitive and behavioral screening tools, beyond the ECASc and ALS-CBS, to evaluate both their reliability and validity. It was critical of the methods used to generate cutoff scores as part of the risk of bias and study quality assessment. However, this review did not evaluate the effect of age and education on scores on screening tools and how cutoff scores may vary depending on these factors.

This review excluded test-retest reliability studies where test sessions were more than two weeks apart (20). In such cases the mean duration between test sessions was often around six months (55). While this may reduce practice effects studies may instead be measuring sensitivity to progressive deterioration.

The current review assumed equivalence of the screening measures in different languages. Given that some cognitive items were altered to be culturally suitable, adapted cognitive screening tools may have functioned differently to the original.

All but one study (26) included in this review used healthy control group data for ROC curve analysis in order to evaluate the sensitivity and specificity of screening tool cutoff scores. These multiple studies are, therefore, informative when trying to identify impairment in pwALS when compared to the normal population. The comparison of pwALS with another clinical group (e.g., Alzheimer’s disease; (26)) addresses a different diagnostic question and data on sensitivity and specificity must be examined in that context rather than in the context of distinguishing pwALS from healthy controls.

The conclusion of this review is that the ECASc remains the most suitable tool for assessing cognitive impairment in ALS because of its suitability to the population and the extensive evidence of its reliability (internal consistency and inter-rater reliability) and validity. For behavioral assessment, the evidence examined here suggests that the BBI or ALS-FTD-Q are the most suitable for clinical and research use, with the BBI being the most thorough and ALS-FTD-Q being briefer. Therefore, this systematic review recommends that clinicians and researchers consider the use of the ECASc to measure cognitive change in pwALS. When assessing behavioral change in pwALS clinicians and researchers should weigh up the need for a more comprehensive as opposed to a briefer assessment and, if opting to choose other measures of ALSci and ALSbi, should appraise themselves of the psychometric properties of the measures, and of the clinical groups in which they have been validated, before adopting them for use.

## Supplementary Material

Supplemental Material
