# Supplementary material for: What is the extent of reliability and validity evidence for screening tools for cognitive and behavioral change in people with ALS? A systematic review
Source: Amyotroph Lateral Scler Frontotemporal Degener. 2024 Feb 28:1–15. doi: 10.1080/21678421.2024.2314063 (PMC10972547; doi:10.1080/21678421.2024.2314063)
Supplement: Supplemental Material [file IAFD_A_2314063_SM2219.docx]

## Supplementary material for:

**What is the extent of reliability and validity evidence for screening tools for cognitive and behavioural change in people with ALS? A systematic review.**

**Lyndsay Didcote, Silia Vitoratou, Ammar Al-Chalabi & Laura H. Goldstein**

## Data synthesis and extraction

The following sample characteristics were extracted: the screening tool(s) tested; cohort type (pwALS/other patient group/healthy controls); mean (and standard deviation) or median (and interquartile range) age, years of education, disease duration, and ALSFRS-R scores; percentage of the sample that was female; the language in which the research was conducted; sample size; and disease onset site profile of samples.

Reliability data that was extracted related to: internal consistency statistics; item-total correlation; test-retest reliability and inter-rater reliability statistics (intraclass correlations and Cohen’s kappa). Validity data that was extracted specifically included (where available): the cut-off score recommended by the study; sensitivity, and specificity of the cut-off score; area under the curve for distinguishing between cases and controls; structural validity statistics; statistics relevant to convergent validity (e.g., Pearson’s correlations). Sensitivity and specificity values were often reported in the literature, so studies can be compared using these metrics, while few report negative predictive values (NPVs) and positive predictive values (PPVs); therefore, it is not easy to compare studies and draw clear conclusions based on PPVs and NPVs. Thus, only sensitivity and specificity values were extracted.

## Risk of bias and study quality (Main score)

Sample sizes of 30 or more participants used to derive the reported reliability and validity statistics were awarded one point. Sample sizes of below 30 were considered small and were not awarded points. A sample size of at least 30 was preferred as it is considered large enough to rely on Central Limit Theorem and assume that the sampling distribution of the mean approximates a normal distribution (1).One point was awarded if reliability and validity evidence was generated using a sample of ALS participants. Samples that did not comprise ALS participants were not awarded any points.

Two points were awarded if a neuropsychological test battery (a collection of several standardised cognitive/behavioural measures) or clinical diagnosis was used as a gold standard classification of cognitive or behavioural impairment in the process of generating cut-off scores for screening tools. If a widely accepted cognitive/behavioural screening tool, other than an ALS-specific screening tool, was used as a gold standard classification, one point was awarded. No points were awarded if a gold standard was not used to determine cognitive or behavioural status when cut-off scores were generated.

## Risk of bias and study quality for inter-rater reliability studies

For studies that assessed inter-rater reliability, a second risk of bias and study quality score, specific to inter-rater reliability evaluations, was given. As for the main risk of bias and study quality score, inter-rater reliability risk of bias and study quality scores (IRR bias/quality scores) were calculated as total points awarded for each bias assessment criterion over total points available (not all bias assessment criteria were applicable to each study) and are given as a percentage.

IRR risk of bias and study quality scores were generated based on the sample size and whether statistics were generated using an ALS sample. The sample size and ALS sample criteria, and the point allocation method were the same for the IRR risk of bias and study quality score as was used to generate the main risk of bias and study quality score.

IRR risk of bias and study quality was further evaluated by assessing the use of blinding techniques, and random selection of participants where appropriate. If raters were blinded to scores given by other raters, studies were awarded one point as this method minimises the risk of raters influencing each other’s scores. Studies were not awarded points if this blinding method was not in place or was not described in the research article. Where a sub-sample was selected from the main study sample (i.e., only a proportion of the sample was selected) to calculate inter-rater reliability, studies were awarded one point if the sub-sample was randomly selected. Studies were not awarded any points if the sub-sample was not randomly selected or if this information was not provided in the research article.

As some risk of bias and study quality assessment criteria were not relevant to every study included in the review, the total points scored by each study are expressed as a percentage of the total available points for the relevant criteria.

## Registration

This review was not registered and a protocol was not prepared.

## Papers that were discussed among researchers and excluded

| Paper | Reason for exclusion |
| --- | --- |
| Crockford, Kleynhans et al., 2018(2) | Alternate forms rather than test-retest reliability |
| Crockford, Newton et al., 2018(3) | Alternate forms rather than test-retest reliability |
| Christodoulou et al., 2016(4) | Alternate forms rather than test-retest reliability |
| Poletti et al., 2018(5) | Test-retest intervals were longer than 2 weeks apart at 6 months (longitudinal rather than test-retest). |
| Bock et al., 2017(6) | Test-retest intervals were longer than 2 weeks apart at 6.8 months (longitudinal rather than test-retest). |

1. Kwak SG, Kim JH. Central limit theorem: the cornerstone of modern statistics. Korean J Anesthesiol. 2017;70(2):144–56.

2. Crockford C, Kleynhans M, Wilton E, Radakovic R, Newton J, Niven EH, et al. ECAS A-B-C: alternate forms of the Edinburgh Cognitive and Behavioural ALS Screen. Amyotroph Lateral Scler Frontotemporal Degener. 2018;19(1–2):57–64. Available from: https://www.tandfonline.com/action/journalInformation?journalCode=iafd20

3. Crockford C, Newton J, Lonergan K, Madden C, Mays I, O’sullivan M, et al. Measuring reliable change in cognition using the Edinburgh Cognitive and Behavioural ALS Screen (ECAS). Amyotroph Lateral Scler Frontotemporal Degener. 2018;19(1–2):65–73. Available from: https://www.tandfonline.com/action/journalInformation?journalCode=iafd20

4. Christodoulou G, Gennings C, Hupf J, Factor-Litvak P, Murphy J, Goetz RR, et al. Telephone-Based Cognitive-Behavioral Screening for Frontotemporal Changes in Patients with Amyotrophic Lateral Sclerosis (ALS). Amyotroph Lateral Scler Frontotemporal Degener. 2016;17(8):482–8. Available from: https://www.ncbi.nlm.nih.gov/pmc/articles/PMC5356507/pdf/nihms849584.pdf

5. Poletti B, Solca F, Carelli L, Faini A, Madotto F, Lafronza A, et al. Cognitive-behavioral longitudinal assessment in ALS: the Italian Edinburgh Cognitive and Behavioral ALS screen (ECAS). Amyotroph Lateral Scler Frontotemporal Degener. 2018;19(5–6):387–95. Available from: https://www.tandfonline.com/action/journalInformation?journalCode=iafd20

6. Bock M, Duong YN, Kim A, Allen I, Murphy J, Lomen-Hoerth C. Progression and effect of cognitive-behavioral changes in patients with amyotrophic lateral sclerosis. Neurol Clin Pract. 2017;7(6):488–98.
